# Supplementary material for: On the quantification of sample microstructure using single-exposure x-ray dark-field imaging via a single-grid setup
Source: Sci Rep. 2023 Jul 7;13:11001. doi: 10.1038/s41598-023-37334-3 (PMC10329004; doi:10.1038/s41598-023-37334-3)
Supplement: Supplementary file 1 — Supplementary Information. [file 41598_2023_37334_MOESM1_ESM.pdf]

Supplementary Information – On the quantification of sample microstructure using single-exposure x-ray dark-field imaging via a single-grid setup

Ying Ying How[1,*], **David M. Paganin**[1], **and Kaye S. Morgan**[1]

[1]School of Physics and Astronomy, Monash University, Clayton, VIC 3800, Australia
[*]Ying.How1@monash.edu

Supplementary information on the speckle patterns observed from the clumped microspheres in our experiments, together with the relevant figures, are given here. A general experimental guideline on how the imaging system can be optimized is provided here. Quantitative results regarding Fig. 6, Fig. 7 and Fig. 9 in the main text are also provided.

Speckle pattern in the dark-field images

From Fig. 3 in the main text of the paper, we observed that the 6.2 µm sample produced stronger local variations in the change in grid visibility, in the mean (transmission) intensity, and hence the dark-field signal and scattering angle images, compared to the rest of the sample. The speckle pattern observed in the dark-field image of the 6.2 µm sample may be attributed in part to propagation-based phase contrast edge effects, contrast which is evident from the transmission images (see Fig. 3(d), (e) & (f)), increasing with propagation distance. If this were the case, then by removing the propagation-based phase effect, we should retrieve a dark-field signal image with no speckle pattern. This correction can be achieved by dividing the raw data by the simulated intensity of the x-ray wavefield propagated through free space after exiting from the sample (as demonstrated in Groenendijk *et al.*[1]), before performing our method of analysis. Figure S1(b) shows the resulting intensity seen after propagating an x-ray wavefield with uniform intensity through the sample (with thickness shown in Fig. S1(a)) and then an extra distance of 2.8 m, with the free-space propagation performed via the angular-spectrum approach[2] with an assumption that the sample is not attenuating, using $\delta = 2.0463 \times 10^{-7}$ (polystyrene at 34 keV) and $\beta = 0$, where the complex refractive index is $n = 1 - \delta + i\beta$. Figure S1(d) shows the result obtained after dividing the raw sample-grid image (Fig. S1(c)) by the propagated grid-free intensity determined by simulation (Fig. S1(b)). The majority of the fringes formed within the sample itself and near the edges of the glue down the bottom and near the rubber wedges can no longer be seen in Fig. S1(d), indicating that the propagation-based phase effects have been removed.

The dark-field signals extracted from the images taken at a propagation distance of 2.8 m before and after this correction are shown in Fig. S1(e) and (f) respectively. We observed that Fig. S1(f) agrees with Fig. S1(e) except that (f) appears to be very slightly smoother. This not only suggests that the speckle pattern formed in the 6.2 µm sample tube does not originate from the propagation-based phase effects, but it also indicates that our algorithm can still work effectively even in the presence of propagation-based fringes. A similar result is observed from other samples, as shown in Fig. S2.

The presence of clusters and air gaps in the 6.2 µm tube, as described earlier in the paper, can be further justified by examining Fig. S1(c) & (d). Here, we see that some regions in the grid intensity pattern are more blurred out, compared to the rest of the pattern. As expected, this corresponds to the regions with greater magnitude in the thickness image (Fig. S1(a)) and also to the regions with stronger dark-field signal in Fig. S1(e) & (f). It is also worth noting that the clusters formed in the 6.2 µm sample act as additional microstructure in the sample, with a length scale different from the individual microspheres. The discontinuity observed from the dark-field signal produced by the 6.2 µm sample across different propagation distances (see Fig. S3) suggests that the blurring from the extra sample microstructure may be of the same length scale as the grid period, thus causing the grid intensity pattern to distort further, until the cross-correlation analysis could not work properly and potentially also leading to the saturation of the dark-field signal.

A small number of pixels in the 6.2 µm sample tube have shown an increase in visibility, as demonstrated by the dark-field signals detected from these pixels being greater than 1. This may be due to the focusing of x-rays by the air gaps. It may also be due to clumps within the sample that are barely resolved – i.e., not sufficiently smaller relative to the pixel size to produce a dark-field signal, but also not sufficiently well-resolved to produce a visible phase shift in the x-ray wavefield. Another contributing factor may be grid-period-sized phase-induced distortions coming from the barely-resolved sample clumps mentioned above, which may locally change the visibility of the reference intensity pattern.

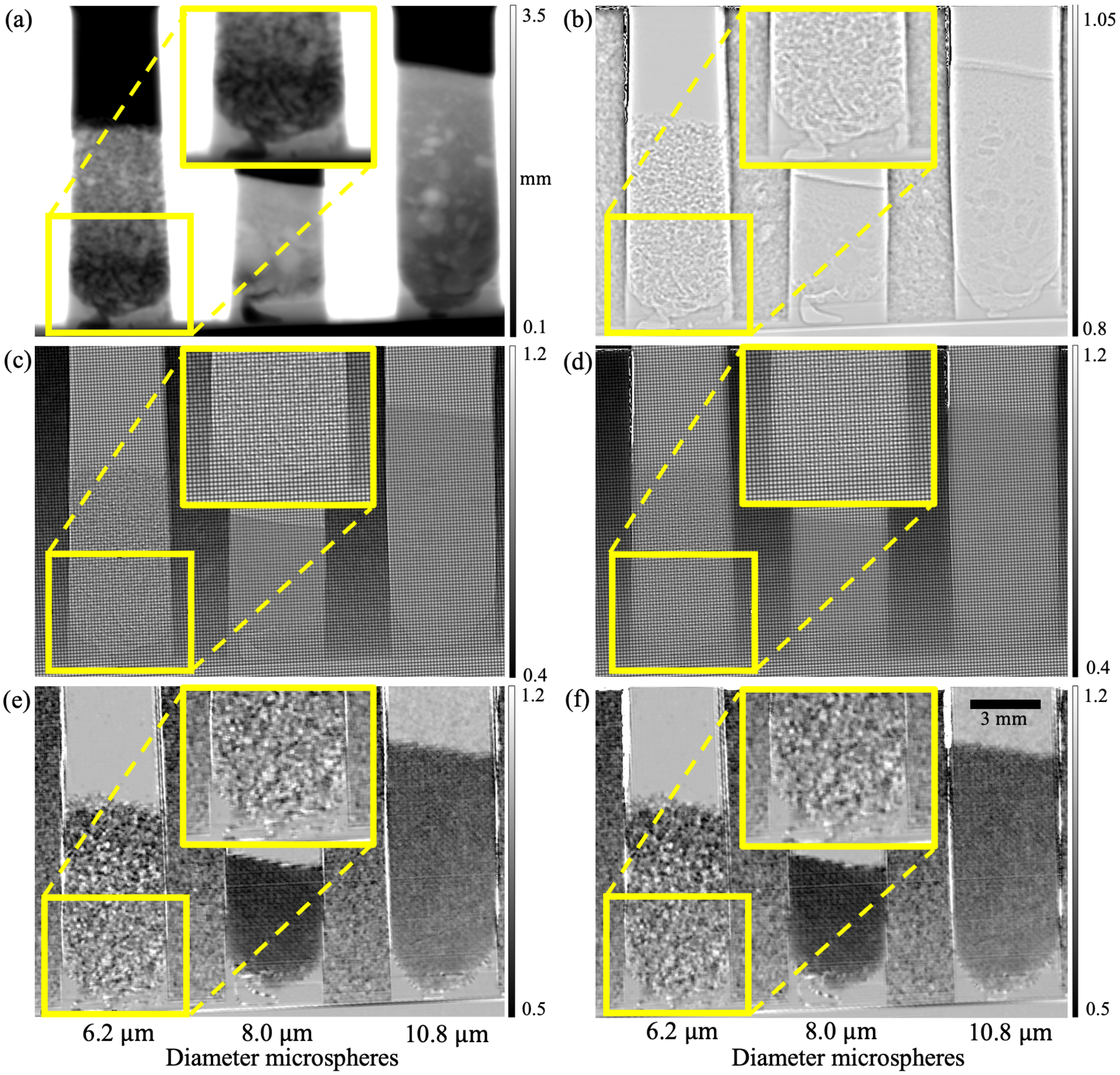

Figure S1. Dark-field retrieval while removing propagation-based phase contrast edge effects. The diameter of the microspheres in each panel (from left to right) is 6.2 µm, 8.0 µm and 10.8 µm. (**a**) Thickness image retrieved via the transport-of-intensity equation (TIE)-based phase retrieval algorithm[3] using the transmission signal extracted from images taken at a propagation distance of 2.8 m. (**b**) Simulated intensity seen after propagating a uniform-intensity x-ray wavefield by 2.8 m, via the angular spectrum approach[2], using the phase information from (a) and assuming the sample is not attenuating. (**c**) The raw sample-grid image at 2.8 m, and (**d**) the same, after dividing out the propagation-based phase contrast edge effects (panel (b)). (**e**) & (**f**) The dark-field signal extracted from panels (c) and (d), respectively. Some artefacts can be observed near the edges of the rubber wedges in (b). The dark-field signals in (e) and (f) are similar, suggesting that the speckle pattern observed in the dark-field-signal image does not originate from the propagation-based phase contrast fringes, but truly from local variations in dark-field. This result also suggests that our algorithm can still extract the dark-field signal accurately, even in the presence of propagation-based phase contrast edge effects.

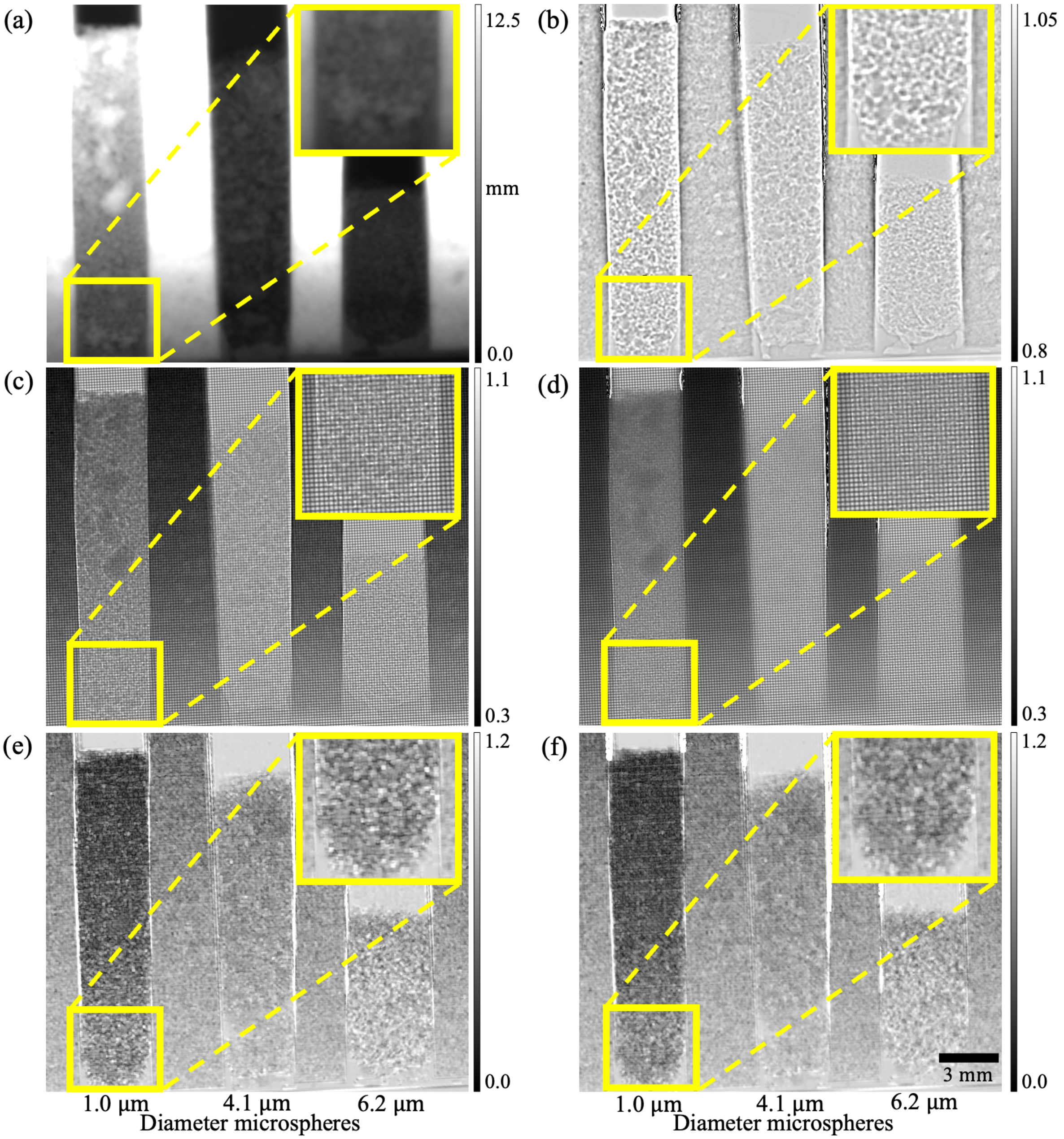

Figure S2. Dark-field retrieval, using an analysis which removes edge effects that are due to propagation-based phase contrast. The diameter of the microspheres in each panel (from left to right) is 1.0 µm, 4.1 µm and 6.2 µm, respectively. (**a**) Thickness image recovered via a phase-retrieval algorithm[3] that is based on the transport-of-intensity equation[4], using the transmission signal extracted from images taken at a propagation distance of 2.8 m. (**b**) Simulated intensity of an x-ray wavefield with uniform intensity, after being numerically propagated 2.8 m via the angular spectrum approach[2], using the phase information from (a) and assuming the sample is not attenuating. (**c**) The raw sample-plus-grid image at 2.8 m, and (**d**) the same, after dividing out the propagation-based phase contrast edge effects (panel (b)). (**e**) & (**f**) The dark-field signal extracted from panels (c) and (d), respectively. Some artefacts can be observed near the edges of the rubber wedges in (b).

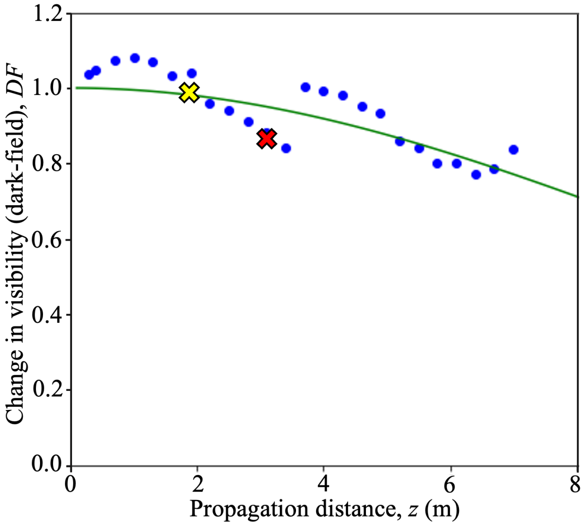

Figure S3. A typical example of the dark-field signal measured as a loss in visibility (at pixel (960, 490), from the 6.2 μm sample) using 24 propagation distances. The red and yellow crosses label the dark-field saturation and maximum-gradient point, respectively, as explained in Fig. 1 of the main text of the paper. One or multiple discontinuities are observed from the dark-field signal measured from this sample across different propagation distances. This may be due to the extra distortion of the grid intensity pattern, contributed by blurring from the extra sample microstructure of the same length scale as the grid period. The scattering angle is extracted from the dark-field signal, measured at the shorter propagation distances to minimise the contributions from the extra sample microstructure.

A general experimental guideline

A general guideline on how the imaging system can be optimized experimentally for different samples is given here. First, we need a sample that contains microstructures that are not resolvable by the imaging system, which means that they are smaller than the spatial resolution of the system. The effective pixel size generally provides a good estimation of the maximum microstructure size that can produce an observable dark-field signal in a specific setup. As demonstrated in this manuscript, a sample with a smaller microstructure size produces a stronger dark-field signal compared to a sample with a larger microstructure size. The sample used in this experiment (with microstructure size ranging from 1-10 µm) provides a good reference on the suitable microstructure size that can generate sufficient dark-field signal in an imaging system with an effective pixel size of 9.8 µm. In terms of the sample material, a suitable sample will be one where the microstructure is made up of material that has a refractive index decrement (δ), which is distinct from that of the surrounding medium, for example, polystyrene and air. In terms of thickness, a sample with a greater thickness generally results in greater scattering. For a sample with a microstructure size of 1-10 µm, a thickness of a few mm is enough to produce a strong dark-field signal.

It is possible to perform an experimental optimization of the setup itself to adjust the sensitivity of the imaging system to the dark-field signal, given the flexibility in our imaging technique. When deciding the grid period, we ideally want to place one grid period over 7-15 pixels so that the grid intensity pattern measured on the detector is well sampled and can be accurately modelled by a sinusoid. The position of the grid relative to the source is not critical as long as the grid intensity pattern measured on the detector has high visibility (as shown in Fig. 1(b)). The propagation distance can then be determined experimentally after introducing the sample. As mentioned in the fourth paragraph of the 'Properties of the technique' section, when imaging at a suitable propagation distance, the grid intensity pattern should still be visible with observable blurring (as shown in Fig. 1(c) & (d)), but not blurred out so much that the grid intensity pattern is hardly visible and resulting in saturated dark-field signal/reaching the 'visibility floor' (as shown in Fig. 1(e) & (f)).

The tunable sensitivity of our imaging system to the dark-field signal via the change in propagation distance allows us to image samples with a range of thicknesses and characteristic sizes. For example, when the sample has a greater thickness or a smaller microstructure size, we can decrease the propagation distance to reduce the blur width and vice versa, as suggested by the optimal propagation distance expression we derived from our model (Eqn. (8)).

Quantitative data for figures

Table S1 shows the fitted value of all parameters in Fig. 6 from the main text of the paper. Tables S2 and S3 show the raw plot data in Fig. 7 and Fig. 9 from the main text of the paper, respectively.

S (μm)	K (rad/$\sqrt{\text{m}}$)	b (m, polystyrene)	b (m, Kapton)	K' (rad/$\sqrt[p]{\text{m}}$)	p
(a) 1.0	0.000405 ± 0.000003	0.00155 ± 0.00004	0.00109 ± 0.00003	0.00044 ± 0.00002	1.927 ± 0.041
(b) 4.1	0.000337 ± 0.000003	0.00088 ± 0.00002	0.00062 ± 0.00001	0.00048 ± 0.00005	1.796 ± 0.052
(c) 6.2	0.000261 ± 0.000007	0.00068 ± 0.00003	0.00048 ± 0.00002	0.0004 ± 0.0001	1.818 ± 0.147
(d) 6.2	0.000263 ± 0.000009	0.00071 ± 0.00004	0.00050 ± 0.00003	0.0003 ± 0.0001	1.960 ± 0.187
(e) 8.0	0.000343 ± 0.000004	0.0007450 ± 0.0000004	0.0005261 ± 0.0000003	0.0010 ± 0.0002	1.506 ± 0.057
(f) 10.8	0.000302 ± 0.000002	0.000614 ± 0.000005	0.000434 ± 0.000004	0.00059 ± 0.00005	1.667 ± 0.036

Table S1. Fitted parameters (within 68% confidence interval) for the orange ($\theta = K\sqrt{T-b}$) and red ($\theta = K'\sqrt[p]{T-b}$) curves from each panel in Fig. 6 of the main text of the paper. The background thickness, b of Kapton is calculated by dividing b of polystyrene by 1.4161 ($\beta_{Kapton}/\beta_{polystyrene}$).

S (μm)	K (rad/$\sqrt{\text{m}}$)	$u(K)$ (rad/$\sqrt{\text{m}}$)	$1/K^2(\times 10^7)$ (m/rad^2)	$u(1/K^2)(\times 10^7)$ (m/rad^2)
1.0	0.00041	0.00007	0.61	0.22
4.1	0.00034	0.00004	0.88	0.23
6.2	0.00026	0.00008	1.44	0.83
8.0	0.00034	0.00005	0.85	0.24
10.8	0.00030	0.00002	1.10	0.17

Table S2. The raw data, including the uncertainties, for the plots in Fig. 7 of the main text of the paper.

S (μm)	Theoretical z_{opt} (m) (green)	Experimental z_{opt} (m) (red)	Theoretical prediction (m)
1.0	0.33	1.00	0.24 - 1.18
4.1	1.22	1.90	0.90 - 2.29
6.2	1.81	2.80	1.43 - 2.88
8.0	1.96	2.50	1.52 - 3.38
10.8	2.35	2.50	1.79 - 4.46

Table S3. The raw data for the plot in Fig. 9 of the main text of the paper.

References

1. Groenendijk, C. F., Schaff, F., Croton, L. C., Kitchen, M. J. & Morgan, K. S. Material decomposition from a single x-ray projection via single-grid phase contrast imaging. *Opt. Lett.* **45**, 4076–4079 (2020).
2. Paganin, D. M. *Coherent X-ray Optics* (Oxford University Press, Oxford, 2006).
3. Paganin, D., Mayo, S. C., Gureyev, T. E., Miller, P. R. & Wilkins, S. W. Simultaneous phase and amplitude extraction from a single defocused image of a homogeneous object. *J. Microsc.* **206**, 33–40 (2002).
4. Teague, M. R. Deterministic phase retrieval: a Green's function solution. *J. Opt. Soc. Am.* **73**, 1434–1441 (1983).
